# Resistive Switching Memory of TiO_2_ Nanowire Networks Grown on Ti Foil by a Single Hydrothermal Method

**DOI:** 10.1007/s40820-016-0116-2

**Published:** 2016-11-21

**Authors:** Ming Xiao, Kevin P. Musselman, Walter W. Duley, Norman Y. Zhou

**Affiliations:** 1 Centre for Advanced Materials Joining, University of Waterloo, Waterloo, ON N2L 3G1 Canada; 2 Waterloo Institute of Nanotechnology, University of Waterloo, Waterloo, ON N2L 3G1 Canada; 3 Department of Mechanics and Mechatronics Engineering, University of Waterloo, Waterloo, ON N2L 3G1 Canada; 4 Department of Physics and Astronomy, University of Waterloo, Waterloo, ON N2L 3G1 Canada

**Keywords:** TiO_2_ nanowire networks, Resistive switching memory, Ti foil, Hydrothermal process, Al electrode

## Abstract

The resistive switching characteristics of TiO_2_ nanowire networks directly grown on Ti foil by a single-step hydrothermal technique are discussed in this paper. The Ti foil serves as the supply of Ti atoms for growth of the TiO_2_ nanowires, making the preparation straightforward. It also acts as a bottom electrode for the device. A top Al electrode was fabricated by e-beam evaporation process. The Al/TiO_2_ nanowire networks/Ti device fabricated in this way displayed a highly repeatable and electroforming-free bipolar resistive behavior with retention for more than 10^4^ s and an OFF/ON ratio of approximately 70. The switching mechanism of this Al/TiO_2_ nanowire networks/Ti device is suggested to arise from the migration of oxygen vacancies under applied electric field. This provides a facile way to obtain metal oxide nanowire-based ReRAM device in the future.

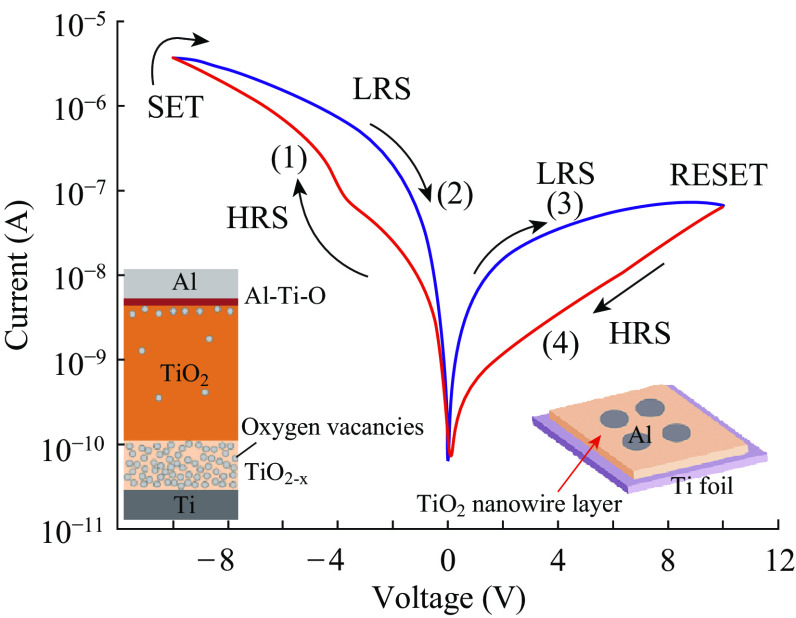

## Highlights

- TiO_2_ nanowire networks were grown on Ti foil by a one-step hydrothermal method.
- Obtained Al/TiO_2_ nanowire networks/Ti devices showed forming-free resistive switching behavior. Good retention and endurance performance was achieved for the fabricated devices.
- Switching mechanism is due to migration of oxygen vacancies under electric field.

## Introduction

Resistive switching random access memory (ReRAM) utilizing an electric-field-induced resistance switching phenomena has attracted great attention for next-generation nonvolatile memory due to its advantages of simple sandwich structure of metal/insulator/metal, high storage density, and fast operation speed [1, 2]. Among different metal oxide materials that demonstrate potential for ReRAM, including NiO [3, 4], TiO_2_ [1, 5, 6], ZnO [2, 7, 8], VO_2_ [9], Ta_2_O_5_ [10, 11], CuO [12, 13], WO_3_ [14], etc., TiO_2_ nanomaterial-based memory has been widely studied due to its ease of fabrication [1, 15] and its ability to demonstrate both unipolar [16, 17] and bipolar [18–20] resistive switching behavior.

Compared to TiO_2_ thin films used for ReRAM [6, 19–22], few studies based on one-dimensional TiO_2_ nanomaterials for ReRAM have been reported. It was recently shown that a single TiO_2_ nanowire-based resistive switching device demonstrated multilevel memory behavior [23, 24]. But the fabrication process of Au electrodes bridging a single nanowire required costly and time-consuming electron-beam lithography. Therefore, a facile way to fabricate TiO_2_ nanowire-based ReRAM is required. Furthermore, TiO_2_ nanorod [25, 26] and nanotube [27] arrays grown on fluorine-doped tin oxide (FTO) glass substrate by hydrothermal synthesis were also employed in resistive switching memory devices, however transparent conductive glass was required as a substrate. It was recently reported that TiO_2_ nanowire networks could be grown directly on Ti foil via a hydrothermal method [28–31] or oxidation process [32, 33], and the applications of these nanowires in dye-sensitized solar cells [29, 30] and field emission [32] were investigated. But the suitability of these TiO_2_ nanowires for ReRAM devices and the corresponding switching mechanism has not been reported yet.

In this paper, TiO_2_ nanowire networks were directly grown on Ti foil by a hydrothermal method and their resistive switching behavior was investigated. Since the Ti foil serves both as the source of Ti during the synthesis of the TiO_2_ nanowire, as well as a bottom electrode for the device, preparation of the device is straightforward, cost effective and highly reproducible. Notably, the electrical contact between the nanowires and the bottom metal substrate is ensured. According to the current–voltage (*I*–*V*) measurements of the fabricated Al/TiO_2_ nanowire networks/Ti device, a switching mechanism based on the migration of oxygen vacancies is proposed. The reliability of the fabricated device was examined by studying its retention and endurance performance.

## Materials and Methods

The synthesis process of TiO_2_ nanowire networks on Ti foil is referred to [28, 29]. Briefly, a piece of Ti foil with a dimension of 1.5 × 3.0 cm × 0.127 mm (Sigma Aldrich) was ultrasonically cleaned in acetone, isopropanol and Milli-Q water for 10 min in sequence and then placed against the wall of a 125 mL Teflon-lined stainless steel antoclave filled with 40 mL of 1 M NaOH aqueous solution. Then, the sealed autoclave was put into an oven at a temperature of 220 °C for 20 h. Next the Ti foil covered with nanowires was taken out of the autoclave and immersed in 50 mL of 0.6 M HCl solution for 1 h to exchange Na^+^ with H^+^. Finally, the foil was annealed inside a furnace at 500 °C for 3 h in air to transform the H_2_Ti_2_O_5_·H_2_O nanowires to anatase nanowires. The color of the foil turned white after the calcination process.

During device fabrication, the top electrode was prepared by depositing an Al layer with a thickness of 150 nm through a shadow mask having circular holes (1 mm in diameter) using e-beam evaporation process (Intelvac e-beam evaporation system). The pressure was <4 × 10^−6^ Torr, and the deposition rate was 1 Å s^−1^. Electrical measurements were performed using a Keithley 2602A source-meter at ambient conditions. The bias voltage was applied to the top Al electrode, and the Ti foil was grounded during electrical measurement.

For the characterization of the TiO_2_ nanowires, a field-emission scanning electron microscope (FESEM, LEO-1550) was used to check the surface morphology. Transmission electron microscopy (TEM, JEOL 2010F) was used to examine the structure and crystalline defects of TiO_2_ nanowires. X-ray diffraction analysis (XRD, PANalytical X’pert PRO MRD) and Raman analysis (Reinshaw micro-Raman spectrometer) were used to identify the crystal structure and phase, respectively. Furthermore, X-ray photoelectron spectroscopy measurement (XPS, Thermo VG Scientific ESCLab 250) was carried out to examine the surface chemical states of the nanowires.

## Results and Discussion

### Characterization of TiO_2_ Nanowire Networks

**Fig. 1:**
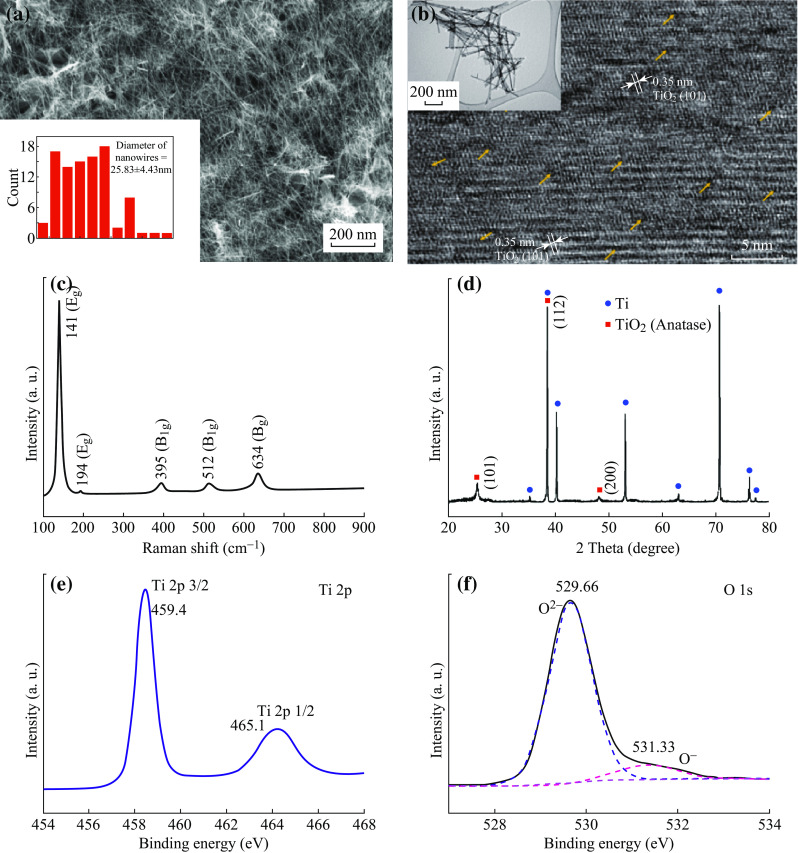
Characterization of TiO_2_ nanowires. **a** Top view SEM image (*inset*, statistical summary of diameters of ~ 100 nanowires); **b** HRTEM image, *yellow arrows* indicate the crystalline defects (*inset*, TEM image of TiO_2_ nanowires); **c** Raman spectrum; **d** XRD results; **e** Ti 2p XPS spectrum; and **f** O 1s XPS spectrum

The SEM image illustrated in Fig. 1a demonstrates a network of TiO_2_ nanowires with lengths of several micrometers from the top view. The statistical summary shows that the nanowires have an average diameter of 26 ± 4 nm. The uniformity of the diameter of the nanowires indicates that the growth occurred predominantly through epitaxial addition of growth units to the tips [29]. During the hydrothermal process, the TiO_2_ nanowires grow perpendicularly to the substrate first and then the tips of nanowires appear to bend and stick together to form a network of nanowires [29]. Therefore, the top Al electrode, as deposited, makes heterogeneous contact with the nanowires, and the *I*–*V* characteristics then reflect the average contact with a large number of individual nanowires. TEM image of the TiO_2_ nanowires (Fig. 1b) shows the (101) plane of anatase in addition to many crystalline defects. These defects could have a significant effect on the resistive switching behavior of the fabricated devices as discussed later in this paper.

A room-temperature Raman spectrum of TiO_2_ nanowires in Fig. 1c shows peaks at 141, 194, 395, 512, and 634 cm^−1^. These peaks are characteristic of the anatase phase. The peaks at 141, 194, and 634 cm^−1^ are assigned to the *E*_g_ modes, while the other two peaks at 512 and 395 cm^−1^ are assigned to the *B*_1g_ modes in TiO_2_ [34]. The XRD characterization results in Fig. 1d further confirm the phase of the TiO_2_ nanowires, as the peaks of (101), (112), and (200) planes of anatase in agreement with the standard spectrum (JCPDS No. 21-1272). It should be noted that one of the anatase peaks at 38.57^°^ overlapped with the peaks of the Ti foil (JCPDS No. 44-1294).

Moreover, the surface chemical states of the TiO_2_ nanowires were analyzed by XPS. Figure 1e shows peaks at binding energies of 459.4 and 465.1 eV, which can be assigned to Ti 2p_3/2_ and 2p_1/2_, respectively. These are typical XPS spectra of Ti^4+^ in TiO_2_. The signal from Ti^3+^ is too small to be detected. Furthermore, two Gaussian peaks are observed in the fit to the O 1s spectrum (Fig. 1f). The binding energy at 529.66 eV is assigned to the O^2–^ bond in TiO_2_ while the binding energy at 531.33 eV can be attributed to oxygen vacancies in TiO_2_ [27]. XPS scans show that the synthesized TiO_2_ nanowires contain locally distributed oxygen vacancies, in agreement with the high-resolution TEM (HRTEM) result in Fig. 1b.

### Electrical Performance Evaluation

#### Resistive Switching Characteristics

**Fig. 2:**
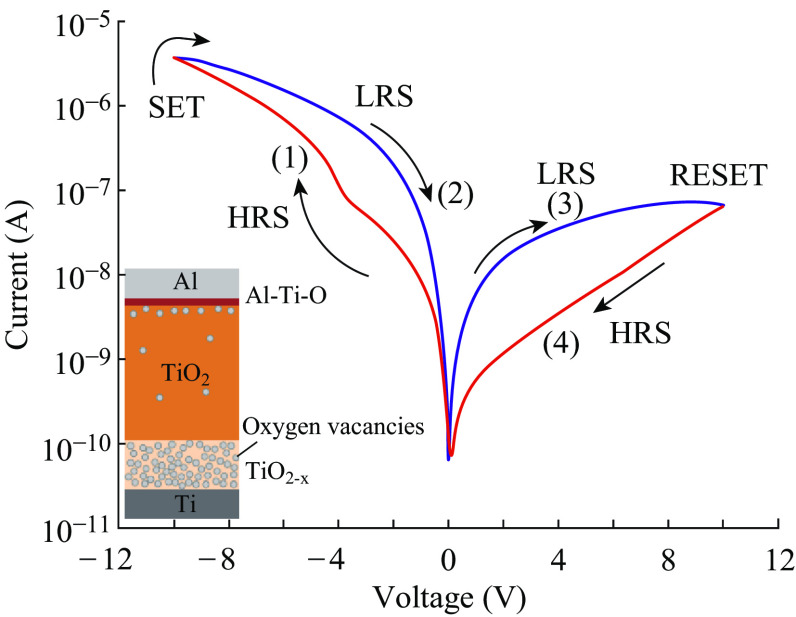
*I*–V characteristic curves of the Al/TiO_2_ nanowire networks/Ti device (*inset*, schematic diagram of the device in the pristine state)

The resistive switching behavior was examined by applying the voltages as follows: 0 V → −10 V → 0 V → 10 V → 0 V with a sweeping rate of 0.1 V s^−1^. The *I*–*V* characteristic curve illustrated in Fig. 2 demonstrates a typical bipolar switching behavior. The Al/TiO_2_ nanowire networks/Ti device was initially in the high-resistance state (HRS). During the application of the negative sweep from 0 to −10 V, the negative current increased gradually, and the device switched to low-resistance state (LRS, ON state) (SET process). The device maintained the LRS during the forward voltage sweep but switched back to the HRS during the voltage sweep back from 10 to 0 V (RESET process). Notably, the resistive switching behavior is obtained without an initial electroforming process, which was normally required for devices consisting of TiO_2_ thin films [6, 35, 36]. This is expected to be due to the high concentration of defects (oxygen vacancies) in the TiO_2_ nanowires after the synthesis process, as seen in the HRTEM image in Fig. 1b and the O 1s XPS spectrum in Fig. 1f. Forming-free resistive switching behavior has also been observed with metal oxide materials containing a large defect concentration [12, 37, 38]. However, some devices require forming treatments, such as hydrogen annealing [39] and Ar^+^ irradiation [40] to generate an oxygen vacancy layer to enable or enhance the resistive switching characteristics. This electroforming-free characteristic is attractive for ReRAM since it would simplify the memory operation and enable higher density memory devices [41].

It should be noted that this resistive switching behavior is quite different from Ref. [17], which shows that Ti/TiO_2_ film/Al structures display linear *I*–V characteristic curves due to the Ohmic–Ohmic contact combination. The difference is expected to be ascribed to the reactions at the Ti/TiO_2_ and Al/TiO_2_ interfaces during the synthesis of the TiO_2_ nanowire networks and the deposition of Al layer. In general, the Ti layer is regarded as a chemically reactive contact that will reduce the TiO_2_ and create a locally high concentration of oxygen vacancies near the Ti/TiO_2_ interface [19, 42, 43]. The formation reaction of oxygen vacancies is expressed in the Kröger–Vink notation [44] as

1 $$\text{O}_{\text{O}}^{ \cdot \cdot } \to \text{V}_{\text{O}}^{ \cdot \cdot } + 2\text{e}^{ - } + \frac{1}{2}\text{O}_{2}$$

where $\text{O}_{\text{O}}^{ \cdot \cdot }$ is the oxygen on the TiO_2_ lattice, and $\text{V}_{\text{O}}^{ \cdot \cdot }$ is a positively charged oxygen vacancy. The generation of oxygen vacancies near the interface between Ti and TiO_2_ nanowires is enhanced during the calcination process due to an increase in the diffusion of Ti atoms into the TiO_2_ layer at high temperature. Therefore, a nonstoichiometric TiO_2-*x*_ (*x* > 0) layer with a high concentration of oxygen vacancies would be formed between the Ti foil and TiO_2_ nanowires. Oxygen vacancies in TiO_2_ act as n-type dopants with shallow donor states below the conduction band and would transform the insulating metal oxide into an electrically conductive semiconductor [5, 6]. In addition, oxygen vacancies in TiO_2_ exhibit higher mobility than metal interstitials at room temperature, so that a number of oxygen vacancies in TiO_2_ are expected to dominate resistive switching behavior [15]. The distribution of oxygen vacancies in the TiO_2_ nanowire layer is expected to be uniform above the Ti/TiO_2_ interface [39]. On the other hand, during the deposition of the Al layer, the high oxygen affinity of Al results in Al reacting with TiO_2_, forming an interfacial insulating Al–Ti–O layer [1, 18, 45, 46]. Consequently, oxygen vacancies are expected to be generated underneath the interfacial layer according to Eq. 1, although a much smaller concentration of vacancies is expected compared to the Ti/TiO_2_ interface [6]. These interfaces in the pristine state are illustrated schematically in the inset of Fig. 2. The different concentrations of oxygen vacancies distributed at the Al/TiO_2_ interface and Ti/TiO_2_ interface result in asymmetric barriers for charge transport, which plays an important role in the switching behavior of the device.

**Fig. 3:**
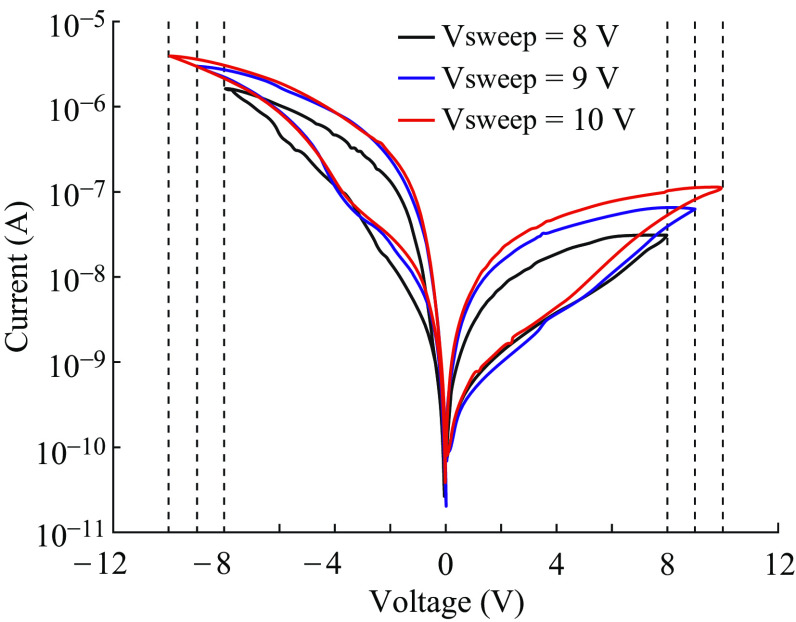
*I*–V characteristic curves of Al/TiO_2_ nanowire networks/Ti device under different sweeping voltages

Furthermore, Fig. 3 shows *I*–V characteristic curves under different sweeping voltages and displays similar bipolar resistive switching behavior in spite of difference in the achieved SET and RESET currents. These results highlight the repeatability of the Al/TiO_2_ nanowire networks/Ti device. Asymmetrical or self-rectifying resistive switching can be seen in both Figs. 2 and 3. The origin of this self-rectifying property can be attributed to effect of the Al–Ti–O layer on the migration of oxygen vacancies as discussed in the next section.

#### Switching Mechanism Analysis

**Fig. 4:**
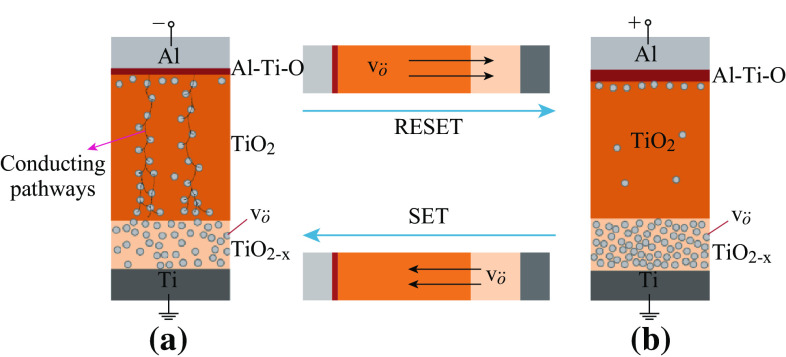
Schematic illustration of **a** SET and **b** RESET process of the Al/TiO_2_ nanowire networks/Ti device

Based on the discussed *I*–V characteristic results, the resistive switching mechanism of the Al/TiO_2_ nanowire networks/Ti device is proposed. As explained, the concentration of oxygen vacancies at the Al/TiO_2_ interface and Ti/TiO_2_ interface is different. These oxygen vacancies are mobile under external electrical field [47]. When the Al top electrode is under a negative bias, the oxygen vacancies in the pristine state (Fig. 4b) migrate towards the top electrode, forming pathways with high electrical conductivity. It is expected that the geometry of the confined TiO_2_ nanowire provides a large surface area and a direct pathway for the migration of oxygen vacancies for stable switching behavior [12]. Simultaneously, electrons would be injected from the Al electrode and drift to the bottom Ti electrode. Once one or more conductive pathways are formed from the top electrode to bottom electrode, the device is switched ON, as illustrated in Fig. 4a. Moreover, some oxygen vacancies may accumulate at the Al–Ti–O layer, which functions as an insulating layer to inhibit out-diffusion of oxygen [45]. This insulating layer may play an important role in the switching behavior since the formation and dissociation of this layer is expected to be closely related to the migration of oxygen vacancies under electric field [1, 46]. Under negative bias, the migration of oxygen vacancies to the Al electrode may result in the partial dissolution of this insulating layer, whereas this layer would be widened when the Al top layer is under a positive bias.

In the ReSET process, the oxygen vacancies in the TiO_2_ matrix are repelled towards the bottom electrode, leading to the recovery of a higher concentration of oxygen vacancies near the Ti bottom layer, and widening of the Al–Ti–O layer as oxygen vacancies drift away from this layer [6]. The presence of a potential barrier in the Al/Al–Ti–O interfacial layer would suppress electron tunneling through the interface and inhibit the formation of conducting channels. Conversely, as oxygen vacancies migrate to the top Al electrode under an applied negative bias, the interfacial layer begins to thin, increasing the probability of electron tunneling and enhancing the formation of conductive channels. As a result, the final current flowing at +10 V is much less than that at −10 V. This is characteristic of asymmetrical or self-rectifying resistive switching behavior, as seen in Figs. 2 and 3. To verify the source of the self-rectifying performance, an identical device was measured without the top electrode, that is, the probe tip (which is made of tungsten) is directly in contact with the top surface of the TiO_2_ nanowire layer. We found the bipolar resistive switching performance existed for the device as well, but no self-rectifying feature was observed.

**Fig. 5:**
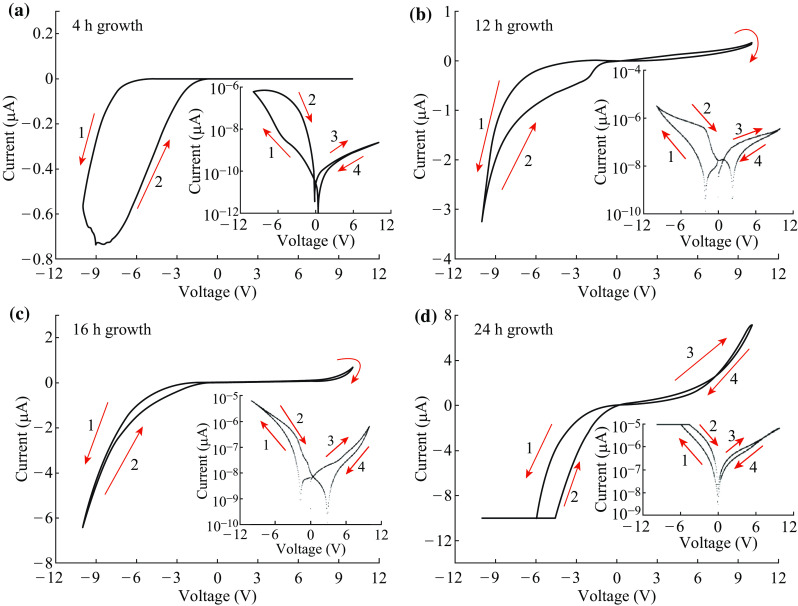
*I*–V characteristic curves of the Al/TiO_2_ nanowire networks/Ti device with different thicknesses of the nanowire layers via the control of the hydrothermal growth time. **a** 4 h growth time, **b** 12 h growth time, **c** 16 h growth time, and **d** 24 h growth time

Further insight into the role of the Al–Ti–O interfacial layer on the resistive switching performance can be obtained by investigating the effect of the thickness of the TiO_2_ nanowire network. This thickness can be varied by changing the hydrothermal growth time. The effect of different growth times (different thickness) on the *I*–V characteristics is illustrated in Fig. 5. These data can be compared with those in Fig. 2 which was obtained for a hydrothermal growth time of 20 h. A self-rectifying resistive switching response was also observed for growth time of 4, 12, and 16 h in addition to 20 h, and the rectification ratio diminishes as the growth time is increased. As discussed, the interfacial Al–Ti–O layer plays an important role in determining the asymmetrical response. The relative effect of this component increases with decreasing thickness of the TiO_2_ nanowire layer so that an increase in growth time to 24 h results in bipolar resistive switching with no obvious self-rectifying feature. The data in Fig. 5a suggests that self-rectifying resistive switching of our Al/TiO_2_ nanowire networks/Ti could, after further optimization, be used to mitigate sneak-current issues in the crossbar-based integration system for ReRAM devices [8].

**Fig. 6:**
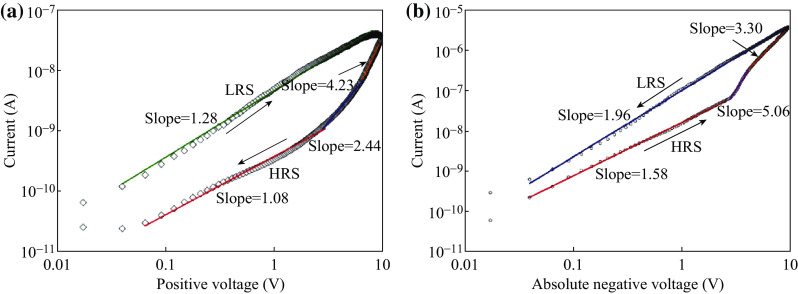
*I*–V characteristic curves under positive **a,** and negative **b** sweeping voltages on a double-logarithmic scale

In order to understand the conduction mechanism of the fabricated Al/TiO_2_ nanowire networks/Ti device, the *I*–V curves in Fig. 2 were fitted on a double-logarithmic scale, as shown in Fig. 6. The overall curve is in good agreement with the trap-associated space charge limited current (SCLC) theory [12, 18, 45]. For the positive sweeping (Fig. 6a), the LRS follows an Ohmic conduction with a slope of ~1, which is consistent with the presence of conductive pathways formed by the migration of oxygen vacancies in the device after the SET process [39]. The *I*–V characteristics in the HRS consist of three regions: the Ohmic region (*I–V*) with a slope of ~1 at low bias, the Child’s square law region *(*I* ~ *V* ^2^) with a slope of 2.44 at higher bias and a region with rapidly changing current near the RESET point (slope: 4.23). The higher slope (>2) compared with the Child’s law in which the current is proportional to the square of the voltage might be due to the expected variation in thickness of the insulating Al–Ti–O layer. This large slope can also be found in similar ReRAM devices in which Al acts as an electrode [18, 45, 48].

The *I*–V characteristic curve for the negative voltage in Fig. 6b also demonstrates SCLC-like behavior but the fitted slope values in different regions are generally larger than those under positive voltage sweeping. This could be due to the concentration gradient of oxygen vacancies that exists in the pristine state (high concentration of vacancies at the bottom Ti/TiO_2_ interface and low concentration underneath the Al–Ti–O layer), which would lead to diffusion of these oxygen vacancies. The diffusion combined with the drift of the oxygen vacancies under an applied negative bias could lead to accelerated migration of vacancies, resulting in higher slopes when transitioning from the HRS to LRS, as compared to the transition from LRS to HRS under positive sweeping. Furthermore, dissociation of the insulating Al–Ti–O layer due to migration of oxygen vacancies under negative bias also decreases the overall resistance of the device, which would contribute to higher values of the slopes. This SCLC-like behavior for both positive and negative sweeping voltages can also be found with increasing sweeping cycles of the device.

The above analysis indicates that Al/TiO_2_ nanowire networks/Ti device as fabricated exhibits a similar *I*–V response and switching mechanism as that seen in devices using a uniform TiO_2_ layer coated with an Al electrode. Such devices were fabricated by time-consuming and costly reactive sputtering [18] or plasma-enhanced atomic layer deposition [1, 45, 46, 48, 49] processes. Therefore, our results indicate that the TiO_2_ nanowire networks grown on Ti foil by a single-step hydrothermal process have potential in the application of ReRAM devices.

#### Endurance and Retention Study

**Fig. 7:**
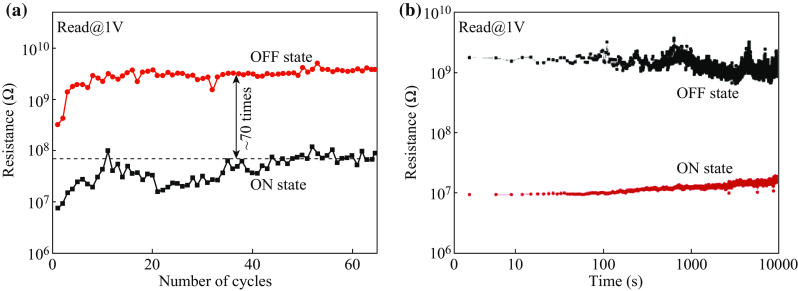
**a** Endurance and **b** retention performance of the Al/TiO_2_ nanowire networks/Ti device

To determine the electrical stability of the fabricated Al/TiO_2_ nanowire networks/Ti device, an endurance study was performed by applying a cycling sweeping process. The results illustrated in Fig. 7a show that the resistance for the OFF state remains stable beyond 60 cycles, while the resistance for the ON state undergo a fluctuation. Nevertheless, the calculated OFF/ON resistance ratio is around 70, large enough to serve as a feasible memory element in ReRAM. Study of endurance under pulsed operation is planned for future work, together with characterization of the device in relation to stability. Furthermore, a data retention test was performed by examining the resistance change with a reading voltage of 1 V for a long period of time after switching the device to ON and OFF states at −10 and 10 V, respectively. The retention results for the ON and OFF states in Fig. 7b demonstrated no remarkable degradation up to 10^4^ s with a high resistance ratio, confirming the nonvolatile nature of the device. The endurance and retention results emphasize good stability of the fabricated Al/TiO_2_ nanowire networks/Ti device for future use as ReRAM.

## Conclusions

In summary, electroforming-free bipolar resistive switching behavior was successfully demonstrated in TiO_2_ nanowire networks directly grown on Ti foil by a one-step hydrothermal process. The prepared Al/TiO_2_ nanowire networks/Ti device exhibited reproducible and stable electrical performance with a high OFF/ON ratio that persisted for up to 10^4^ s. We found that the interaction of Ti foil with the TiO_2_ nanowires during the synthesis process results in the generation of large density of oxygen vacancies at the Ti/TiO_2_ interface, which is likely responsible for the forming-free resistive switching behavior. The switching mechanism of the device is proposed to be the migration of oxygen vacancies under electric field. These results provide an easy way to prepare nanowire-based ReRAM devices with good electrical performance.
